# Mechanical Properties of Percutaneous Transluminal Coronary Angioplasty Balloon Catheters: A Bench Study

**DOI:** 10.1007/s10439-026-04067-z

**Published:** 2026-03-22

**Authors:** C. Amstutz, M. Hersberger, N. Fontaine, J. Behr, A. Haeberlin, R. Vogel, A. Zurbuchen, J. Burger

**Affiliations:** 1 School of Biomedical and Precision Engineering, University of Bern, Bern, Switzerland; 2 SMD Swiss Medical Devices, Beringen, Switzerland; 3 Department of Cardiology, Inselspital, Bern University Hospital, University of Bern, Bern, Switzerland; 4 Department of Cardiology, Buergerspital Solothurn, Solothurn, Switzerland

**Keywords:** PTCA balloon catheter, Mechanical properties, Performance characteristics, Pushability, Trackability, Torquability

## Abstract

**Purpose:**

This paper aims to categorize the mechanical properties of eight percutaneous transluminal coronary angioplasty (PTCA) balloon catheters and translate them into clinical needs.

**Method:**

Objective bench tests quantified PTCA balloon catheters' mechanical properties, including tensile strength, kink resistance, bending, torsional behavior, friction, radio-opacity, pushability, and trackability. The results were compared against each other and supplemented by a survey of interventional cardiologists.

**Results:**

Clinical needs with respect to deliverability, dilatation efficiency, and crossability were assessed for each catheter. Results indicate that SC Maverick2 excels in deliverability and crossability but lags in dilatation efficiency compared to NC catheters, though it is rated best to fulfill clinical needs. SC EasyT outperforms Maverick2 in dilatation efficiency but has inferior deliverability, similar to NC catheters.

The NC balloons were comparable. However, Accuforce exhibits highest deliverability, Pantera LEO shows superior dilatation efficiency, NC Emerge shows best crossability, and Sapphire NC24 exhibits lowest performance. According to the survey, Accuforce, Sapphire NC24, and NC Trek are favored over NC Emerge and Pantera LEO. OPN NC offers limited deliverability but can treat lesions where standard NC catheters fail due to its unique rated burst pressure.

Trackability and pushability estimates align better with survey results than those obtained from simulated use-case tests, except for Sapphire NC24 and OPN NC. Torquability measurements show discrepancies with survey ratings, indicating additional influences and rating challenges.

**Conclusion:**

Our study comprehensively analyzes PTCA balloon catheters, emphasizing the importance of integrating mechanical and design attributes throughout development. Performance properties like trackability and pushability were measured, providing unbiased insights for comparison independent of individual practitioner preferences.

## Introduction

**Fig. 1:**
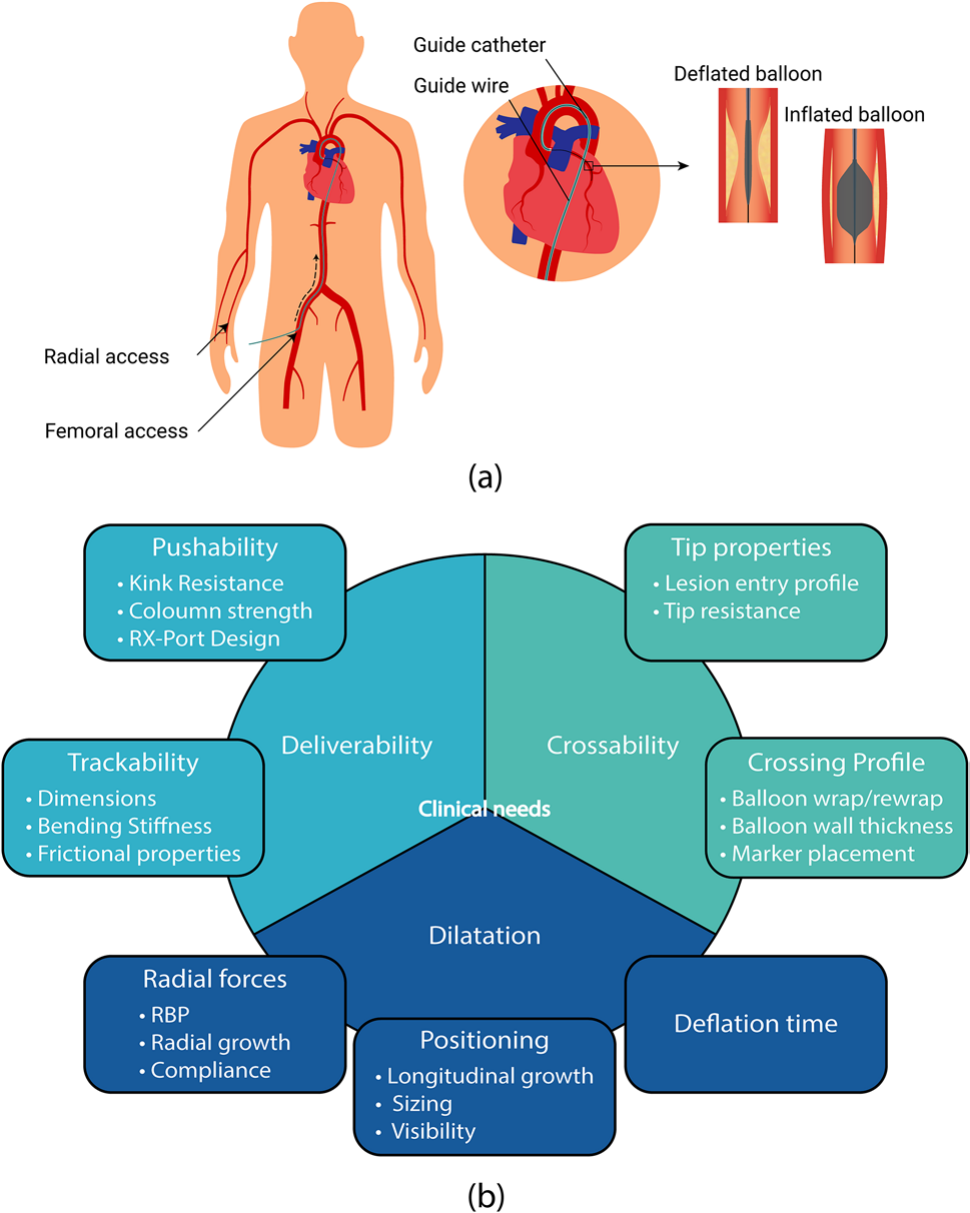
**a** General schematics of a state-of-the-art PTCA intervention. Showing the access site (femoral or radial), the positioning of the catheter in the coronary arteries, and the stenosis before and after balloon inflation. **b** Overview of the clinical needs of a PTCA balloon catheter translated to the design goals

Percutaneous transluminal coronary angioplasty (PTCA) is a minimally invasive procedure to treat stenosed or occluded coronary arteries. The procedure (cf. Fig. 1 a) involves using a catheter with a balloon on the distal end. The balloon is delivered to the diseased coronary artery through a small puncture, typically made in the groin or wrist through the femoral or radial artery, respectively. The catheter is advanced through the aorta into the coronary artery until it reaches the target site. Once in position, the balloon is inflated to widen the diseased vessel and restore blood flow.

Guiding Catheters (GC) and Guide Wires (GW) are essential tools during PTCA procedures [1]. A GC is a large, hollow tube used to access the coronary artery and provide a pathway for the smaller PTCA catheter to be inserted. The tip of the GC is usually placed at the ostium of the diseased coronary artery [2]. The GW is a thin, flexible wire inserted prior to the PTCA catheter, which is used to navigate through the vasculature and guide the catheter to the desired location in the artery. Often, it is necessary to use multiple catheters to treat one or multiple stenoses [3]. The PTCA treatment usually follows a stent placement. Therefore, the GW is usually placed once as a rail for multiple tools during an intervention.

For a successful treatment, a PTCA catheter must comprise three specific properties: (a) Deliverability, the ability to advance the catheter to the desired treatment location; (b) Crossability, the ability to cross even tight lesions; and (c) Dilation, the ability to inflate/deflate the balloon and to dilate the lesion to restore blood flow properly.

Gupta [4] identified the clinical needs: deliverability, crossability, and dilatation. The translation of these clinical needs into the mechanical properties, and subsequently, the design objectives of PTCA balloon catheters, are illustrated in Fig. 1 b).

Despite the existence of PTCA balloon catheters for several decades, there is a limited amount of publicly available literature regarding their mechanical properties. Balloons' mechanical properties and materials have been studied [5–9]. Furthermore, simulations on the fluid-structure interaction of balloon catheters have been performed by Wiesent et al. [10]. Stent systems and the interactions with the balloon have been extensively studied and simulated [11–19]. Barkholt et al. investigated the tip design of PTCA balloon catheters [20]. Experimental pushability and trackability data have been compared to a numerical model by Sirivella et al. [21]. Bending tests on braided GC have been performed by Hijikata et al. [22]. In [23], the performance characteristics of microcatheter were studied. Schmidt et al. developed bench tests to assess the mechanical performance characteristics of drug-eluting stent systems [24]. Wang et al. developed a device to measure the proximal force while inserting catheters into a blood vessel [25]. This device is intended for surgical robots but could be used to classify the performance of a catheter. In [26], an overview of the theoretical background of the mechanical properties of catheters is given.

In a previous study, eight commercially available Rapid eXchange (RX) balloon catheters [27] were investigated regarding their features like tip design, RX-Port design, hypotube design, and the dimensions of the catheter parts. Furthermore, balloon elongation, pull-back forces, and deflation rates have been investigated. Based on this research, further investigations have been carried out, focusing on mechanical properties like tensile, kink, and bending behavior of the catheter parts, friction properties, and use-case properties like deflation time, pushability, trackability, and torqueability. Furthermore, a novel test device has been developed to assess the bending stiffness along the distal part of the catheter.

In order to establish a stronger correlation between this investigation and clinical practice, a comprehensive survey was conducted among interventional cardiologists employing, among others, the Likert scale, thus providing valuable contextual insights. Access to information about the mechanical properties could significantly contribute to advancing development in this field and potentially lead to increased device performance while decreasing catheter costs and periprocedural risks.

## Material and Methods

The same selection of PTCA balloon catheters, GW, and GC as in our previous study [27] has been used during this investigation. The same balloon size with diameter (3mm) and length (20 mm) was chosen.

The selection comprises super high-pressure non-compliant (NC), high-pressure NC, and semi-compliant (SC) balloons. In the following, the eight selected PTCA balloon catheters are listed in alphabetical order, and the used GW and GC:

- Accuforce – Terumo, Tokyo, Japan (Rated Burst Pressure (RBP): 22 [atm], high-pressure NC balloon)
- EasyT– SIS Medical AG, Frauenfeld, Switzerland (RBP: 21 [atm], high-pressure SC balloon)
- NC Emerge – Boston Scientific, Massachusetts, USA (RBP: 20 [atm], high-pressure NC balloon)
- Maverick2 – Boston Scientific, Massachusetts, USA (RBP: 14 [atm], SC balloon)
- OPN NC – SIS Medical AG, Frauenfeld, Switzerland (RBP: 35 [atm], super-high pressure NC balloon)
- Pantera LEO – Biotronik, Berlin, Germany (RBP: 20 [atm], high-pressure NC balloon)
- Sapphire NC24 – OrbusNeich, Hong Kong (RBP: 24 [atm], high-pressure NC balloon, available balloon length: 18 mm)
- NC Trek – Abbott, Illinois, USA (RBP: 18 [atm], high-pressure NC balloon)
- GW: ASAHI SION blue straight (ASAHI INTECC CO. LTD., Tokyo, Japan)
- GC: 5F JL 4.0 GC (Medtronic, Dublin, Ireland)

In total, 15 catheters of each model were used during the investigation. Five catheters were used for each test, and the median and interquartile range (IQR) were evaluated for each measured value. Some catheters were used in multiple investigations if the catheter was not affected by the previous investigations, such as measuring the outer diameter (OD). An overview of the performed tests, the catheters, and the used equipment can be found in Appendix Table 2. Catheters were tested in water at 37 C to replicate conditions akin to the human body, mirroring the body temperature where feasible.

**Fig. 2:**
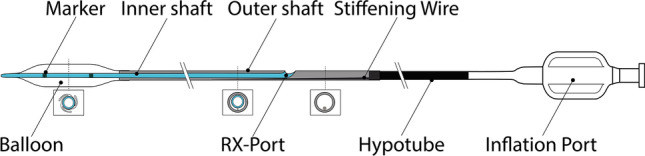
Schematic of an RX-Balloon catheter showing the catheter parts

Fig. 2 shows the schematic of an RX balloon catheter. The catheter is equipped with an inflation port, commonly referred to as the hub, located at the proximal end for connecting a high-pressure syringe (i.e., the indeflator). A metallic hypotube follows the inflation port. At the end of the hypotube, a tapered stiffening wire is attached to gradually reduce the stiffness distally. The RX-Port marks the beginning of the distal part and serves as an exit for the GW. Furthermore, the RX-Port separates the GW lumen defined by the inner shaft (IS) and the inflation lumen defined by the outer shaft (OS). The balloon, containing the radiopaque markers for positioning, is located at the distal end of the catheter.

Manufacturers typically perform a range of design verification tests to ensure the safety and performance of coronary balloon catheters. These include balloon fatigue testing, balloon inflation and deflation time measurement, rated burst pressure (RBP) and balloon failure assessment, crossing profile measurement, dimensional verification, and RX-port tensile testing, as well as package verification, biocompatibility, and hemocompatibility evaluations (ISO 10555-4, ISO 10555-1, ASTM F1980-21, ISO 10993, ISO 16269-8:2004, ISO 13485; list not exhaustive). However, parameters such as trackability and pushability are not required to be disclosed to notified bodies, nor are they reported on product labeling. Instead, manufacturers typically describe performance qualitatively with phrases such as “improved tracking” or “enhanced lesion crossing.” Similarly, friction testing of the inner shaft is not publicly reported.

While these assessments are comprehensive, they are not publicly accessible and rarely incorporate real-world clinician perspectives. The present study addresses this gap by providing an independent comparative analysis across multiple products. In addition to established performance parameters, novel assessments such as friction and distal bending stiffness were introduced, and the evaluation included tensile, kink, and bending properties of catheter components, push- and trackability, torsional stiffness, and radio-opacity. By integrating interventional cardiologist feedback, the analysis aims to better align mechanical performance characteristics with clinical expectations.

### Tensile and kink properties

The tensile properties of the OS, the IS, and the RX-Port were tested on the universal testing machine Shimadzu AGS-X 10 kN (Shimadzu Corporation, Kyoto, Japan). The force $F$ was recorded using a 200 N load cell (accuracy of the measured value of ± 0.5%). The displacement $d$ was recorded by the crosshead position detection (accuracy ± 0.1% indicated value or ± 0.01 mm). Cut pieces with a length of 70 mm are clamped between two grips with a rubber coating to avoid slippage. The gauge length was defined as 35 mm. Based on ISO-253, a 50 mm/min test velocity is used. A wire is inserted over the clamping length to avoid squeezing the tube. The tests were performed in air at 23 C and in a water bath at 37 C. Due to the limited samples, the RX-Port was only tested at the physiological temperature of 37 C. For each test with water, the samples were preheated for five minutes. It was figured that buoyancy forces are generated when moving the clamps out of the water. Therefore, a pre-test was performed with an empty clamp. The force was then subtracted from the measured force. A force-displacement curve is typically transformed into a stress-strain curve to get geometry-independent material properties. In our case, we are interested in the comparative values of the catheters, not the material properties. Therefore, for each sample, a stiffness $k$ is calculated using Eq. 1.

1 $$k\; = \;\frac{\Delta F}{{\Delta d}} \left[ \frac{N}{mm} \right]$$

Furthermore, each sample's force at the yield point is evaluated and compared between the catheter types. On the RX-Port, the fracture force and the maximum displacement are evaluated.

The critical load for the hypotube is considered kinking. Therefore, the hypotubes were cut into pieces with a length of 100 mm. The pieces are clamped between two clamps with a rubber coating and at a distance of 50 mm on the universal testing machine Shimadzu AGS-X. The hypotube is then loaded in the axial direction, and the critical buckling load $F_{crit}$ is measured. The column stiffness $EI_{kink}$ is calculated using the 4th Euler buckling equation Eq. 2.

2 $$EI_{kink} \; = \;\frac{{F_{crit} \cdot \left( {0.5 \cdot L} \right)^{2} }}{{\pi^{2} }} \left[ {Nmm^{2} } \right]$$

### Bending Properties

The bending properties influence the trackability. Using a pulled-bending test (cf. Appendix fig.13), a novel method was developed to assess the bending stiffness over the whole length of the distal part. The test is explained in more detail in the Appendix.

The required force to deflect the catheter by $w = 1.5 mm$ is measured by the universal testing machine Shimadzu AGS-X and a 200N load cell. After reaching this displacement, the catheter is pulled in longitudinal direction while continuing the force measurement, resulting in a force over length $F_{b} \left( l \right)$. Two different pulling velocities $v_{p}$ of 15 mm/s and 50 mm/s were compared. Due to the motorized linear stage, the setup cannot be placed in a water bath, and the tests were conducted in air at 23°C room temperature.

Based on the Euler-Bernoulli beam theory, the bending stiffness ($EI$) was derived. It can be calculated using Eq. 3.

3 $$EI_{PB} \; = \;\frac{{F_{b} \left( l \right) \cdot L^{3} }}{192 \cdot w} \left[ {Nmm^{2} } \right]$$

In addition to the pulled bending, the classic 3-point bending stiffness was measured. Therefore, the catheter was cut into pieces with a length of 70 mm. The IS, OS, RX-Port, and balloon were measured. Furthermore, an OS with an inserted IS shaft was measured to estimate the distal stiffness. The same setup described above has been used without the motorized linear stage. The bending stiffness is calculated using Eq. 4.

4 $$EI_{B} \; = \;\frac{{F_{b} \cdot L^{3} }}{48 \cdot w} \left[ {Nmm^{2} } \right]$$

### Push- and Trackability

The track tester IDTE 1000 (MSI, Phoenix, Arizona, USA) is used to simulate the intervention in the laboratory. The curvature of the coronary arteries is mimicked using an anatomic track model (ASTM F2394). The GW is placed inside the track model, and the GC is placed at the ostium of the coronary model. The setup is shown in Appendix fig. 14 . The PTCA balloon is pushed along the GW through the tortuous vessel path, mimicking the LAD and the Left Circumflex (LCx) artery. The force $F_{track}$, required to move the catheter along the LAD, and LCx is measured. Additionally, the insertion depth is recorded. After insertion of 17 cm, the catheter is retracted. The resulting insertion forces of the catheter models are compared against each other. Furthermore, the area under the curve, which defines the work required for the insertion and pull-back, is evaluated. Moreover, at the end of the LCx, a proximal sensor measures the force on the tip $F_{push}$ at the maximum insertion depth. The pushability is evaluated as the ratio of the $F_{distal}$ and the maximal $F_{proximal}$ in [%]. The tests are carried out in a water bath at 37°C. The water was heated using an ANOVA Precision Cooker (Anova Applied Electronics, Inc, San Francisco, CA, USA) with an accuracy of ± 0.5°C. The GW is replaced after five tests to avoid influences due to the coating wear-off.

In this study, the key parameters influencing the pushability and trackability of PTCA balloon catheters were investigated using multiple linear regression analysis in RStudio 2023.03.0 with R version 4.3.2 (Posit Software, PBC, Boston, Massachusetts, USA). This statistical method allowed for the simultaneous examination of the relationships between several predictor variables and the response variables, pushability, and trackability. It should be noted that pushability defines the force that can be transmitted from the hub to the tip. Therefore, a high value is desired. The trackability, however, is measured as the track work required to insert a catheter into the artificial coronary artery. Lower track work means less resistance. Therefore, a low value is favored. Initially, it was investigated whether there was a significant difference between the trackability measured in the LAD and the LCx based on the Wilcoxon signed-rank test. Although the OPN NC exhibited deviations in BBS, RBP, and CP due to its superhigh-pressure balloon, it was retained in the dataset. This decision was made to preserve the diversity and comprehensiveness of the data. However, a robust linear regression was used for occurrences of extreme observations, resulting in residuals outside of Cook’s distance. Since the RBP is a single value for all catheters, a simulation was performed in R to generate values from a normal distribution based on each balloon’s mean burst pressure (BP) and standard deviation to provide enough variance for the multiple regression analysis. The RBP was converted to the mean BP using Eq. 5 with a $k$-value of 4.278, corresponding to an assumed test set of N = 30 balloons. A standard deviation $s$ of 10% of the RBP was also assumed.

5 $$\begin{gathered} RBP\; = \;\frac{{\sum x_{i} }}{{\underbrace {N}_{{\overline{x}}}}} - k \cdot \sqrt {\frac{{\sum (x_{i} - \overline{x})^{2} }}{{\underbrace {N - 1}_{s}}}} \hfill \\ \hfill \\ \end{gathered}$$

Multicollinearity can negatively affect multiple linear regression analysis since the variables are no longer independent and are, therefore, harder to interpret. To test for multicollinearity the variance inflation factor (VIF) was evaluated. A VIF > 10 was considered too high. To avoid overfitting and to reduce the multicollinearity of the model, the model was reduced by best subset regression in R.

### Friction Characteristics

Various methods have been studied to investigate the friction between the catheter and the GC or GW. The PTCA catheter is usually clamped with a predefined force between two polytetrafluorethylene (PTFE)-coated surfaces and pulled through the clamp to investigate the catheter's coating. The pull force gives information about the coating and the frictional behavior. Takashima et al. [28] described a method to measure the friction between a catheter and a vessel. Furthermore, companies like Alemnis (Thun, Switzerland) developed special microsystems to study the friction of a particular tip against a surface. However, all these methods had certain drawbacks: either the friction partners were different compared to reality, or only the outside of a tube could be measured. Therefore, the device published by P. Wuensche et al. [29] has been recreated to characterize catheter friction. A schematic of this setup is shown and explained further in Appendix fig. 15. The catheter is pulled along the frictional partner for 10 mm with a test velocity of 1 mm/min. The force $F_{z}$, required to pull the catheter, is measured with the universal testing machine Shimadzu AGS-X 10 kN and a 200 N load cell.

The coefficient of friction $\mu$ is measured between a) the outer surface of the hypotube and the inner surface of the GC, b) the outer surface of the GW and the inner surface of the GC’s inner shaft, and c) the left anterior descending (LAD) porcine coronary artery and the outer surface of the OS. It is calculated using Eq. 5 [29].

5 $$\mu_{a, b.c} \; = \;\frac{{\ln \left( {1 + \frac{{\left( {F_{z2} - F_{z1} } \right)}}{{F_{0} }}} \right)}}{\beta }$$

Where the force $F_{z1}$ is the force required to operate the system, $F_{z2}$ is the measured frictional force, and $F_{0}$ is the weight applied to the catheter.

The coefficient of friction $\mu_{a}$ was measured by cutting the GC into pieces with a length of 100 mm. The pieces were bonded onto the wheel $R_{1} { }$ with a diameter of 60 mm, resulting in a contact angle of $\beta = 162.95^\circ$. A weight $F_{0} = 2.562{ }N$ was attached to the inflation port. The weight was chosen to ensure that the hypotube adequately bends and gets in contact with the GC along the whole contact length.

The coefficient of friction $\mu_{b}$ was measured by cutting the inner shafts into pieces with a length of 60 mm. Each inner shaft was bonded onto the wheel $R_{1} { }$ with a diameter of 40 mm, resulting in a contact angle of $\beta = 134.83^\circ$. Since the GW is more flexible than the hypotube, the force was reduced to $F_{0} = 1.07{ }N$.

The coefficient of friction $\mu_{c}$ was measured by dissecting the left anterior descending coronary artery (LAD) (length: 10cm, inner diameter: 2.5mm) from a healthy 120 kg, 6 month-old pig approximately 12 hours post-mortem. Based on pre-trials, the LAD stretches during testing; therefore, no stable plateau for evaluating the coefficient of friction can be reached. As Lin et al. [30] proposed, a pre-stretching of the coronary artery helps avoid the shift of the intima during testing. The coronary artery was cut to a length of 18 mm and stretched to a length of 23 mm. The artery was bonded to the $R_{1}$ with a diameter of 40 mm, resulting in a contact angle of $\beta = 134.82^\circ$.Due to the very flexible tissue, the weight of $F_{0} = 0.4{ }N$ was used to obtain a stable force plateau. The OSs were cut into pieces with a length of 60 mm and affixed to a metal wire measuring 0.16 mm in diameter. This method ensured minimal elongation between the weight attachment and the load cell connection.

Instead of the distilled water, a saline solution at a temperature of 37°C was used.

The frictional coefficient was assessed over a sliding distance of 10 mm. Preliminary tests revealed a decrease in friction when using the guide wire (GW) with more than three catheter types. This frictional change suggested that the hydrophilic coating of the GW might not endure testing with all catheters using a single GW. Consequently, the GW was replaced after evaluating two catheter types with ten samples each. Additionally, the reduction in friction suggested that a bare metal surface performed better than the coating. However, this observation did not hold true for friction within the artery. Moreover, samples of the two catheter types were alternately measured to mitigate the impact of the coating condition.

### Torsional Stiffness

Due to the transition between the stiff hypotube and the flexible distal part, balloon catheters with an RX-Port are not very torquable. However, rotatability or torquability enables another degree of freedom during an intervention when maneuvering the catheter to the desired location. Therefore, this property can be an advantage during a procedure. During the test, the catheter's balloon is clamped to its center using a collet attached to a high-precision torque sensor 8625 4050 (Burster GmbH & Co. KG, Gernsbach, Germany) (accuracy ± 0.1%) (cf. Appendix Fig.16b). Before closing the collet, a stabilization wire is inserted 20 mm into the tip to avoid squeezing the balloon and the IS.

The hub of the catheter is attached to a NEMA11-13-01D-AMT112S stepper motor (CUI INC, Tualatin, Oregon, United States) with a luer connector (cf. Appendix Fig.16b ). The weight of the hub fixation of 42.74 g establishes uniform pre-loading for every catheter longitudinally. Ensuring torsion-free catheter mounting involves initially suspending the fixation freely before anchoring it to the motor.

The length between the collet and the fixation is adjusted for each catheter. Additional information can be found in the Appendix. The rotational behavior of the catheter is studied by rotating the hub by 2 turns over 8s in clockwise and counterclockwise directions. The torque over the whole catheter length is recorded in LabVIEW 2021 (National Instruments, Austin, Texas, United States). By analyzing the slope of the torque vs. angle curve, the catheter’s torquability is quantified.

### Radio-opacity

The balloons are cut from the catheter and placed in the center of a water bath at 23 °C with a depth of 9.75 cm to quantify the radio-opacity of the balloon markers. The balloons are scanned by an ARTIS icono floor (Siemens Healthineers, Erlangen, Germany). For each balloon, a separate image is taken. An aluminum step wedge featuring 11 steps, each spanning a height of 0.5 mm, is positioned alongside the balloons for precise calibration. The step wedge serves as a reference to calibrate each image captured. Using Adobe Photoshop 2022 (San Jose, California, United States), the grayscale values at five points along each step of the wedge were measured. With this calibration, the radio-opacity of the balloon markers can be quantitatively assessed by their grayscale values in mm Aluminum. Values outside the range of the step wedge were linearly extrapolated.

### Survey

A questionnaire was generated using Google Forms to contextualize the bench tests with the clinical work. The questionnaire assessed the perspectives and experiences of interventional cardiologists from Germany and Switzerland. Participants were approached either during conferences or through email invitations.

A total of 22 interventional cardiologists voluntarily responded to the survey. The questionnaire consisted of 25 questions, including both mandatory and optional items. Four of the 25 questions were optional and pertained to the participants' professional backgrounds, such as their position, years of experience, and country of practice.

The questionnaire encompassed eight questions related to the performance of balloon catheters, of which four were designed using a Likert scale to gauge the participants' opinions. Additionally, three multiple-choice questions were included to assess complications associated with balloon catheter usage and the severity of these complications. One ranking question (0, not important to 10, very important) was related to the importance of torquability.

Furthermore, the survey incorporated ten questions about the usage of balloon catheters, covering topics such as the types of lesions treated (measured on a Likert scale and multiple-choice format), treatment approaches for bifurcations and lesions in tortuous vessels (measured on a Likert scale), utilization of the kissing-balloon technique (yes-no question), and participants' preferred equipment. The latter question allowed participants to provide a text-based response, although it was optional.

The questionnaire concluded with three general questions addressing aspects of price and potential improvements. All questions, except the general and text-based response questions, provided an "I don't know" option for participants to select.

During the evaluation process, responses to Likert scale questions were assigned numerical values from 1 to 5 and averaged to determine the participants' average perception or preference. "I don't know" responses were counted but excluded from the averaging process. For multiple-choice questions, the frequency of respective answers was recorded.

The survey data serve as a foundation for this study's subsequent analysis and discussion. The list of questions can be found in the Appendix (Table 3).

## Results

### Tensile and Kink Properties

**Fig. 3:**
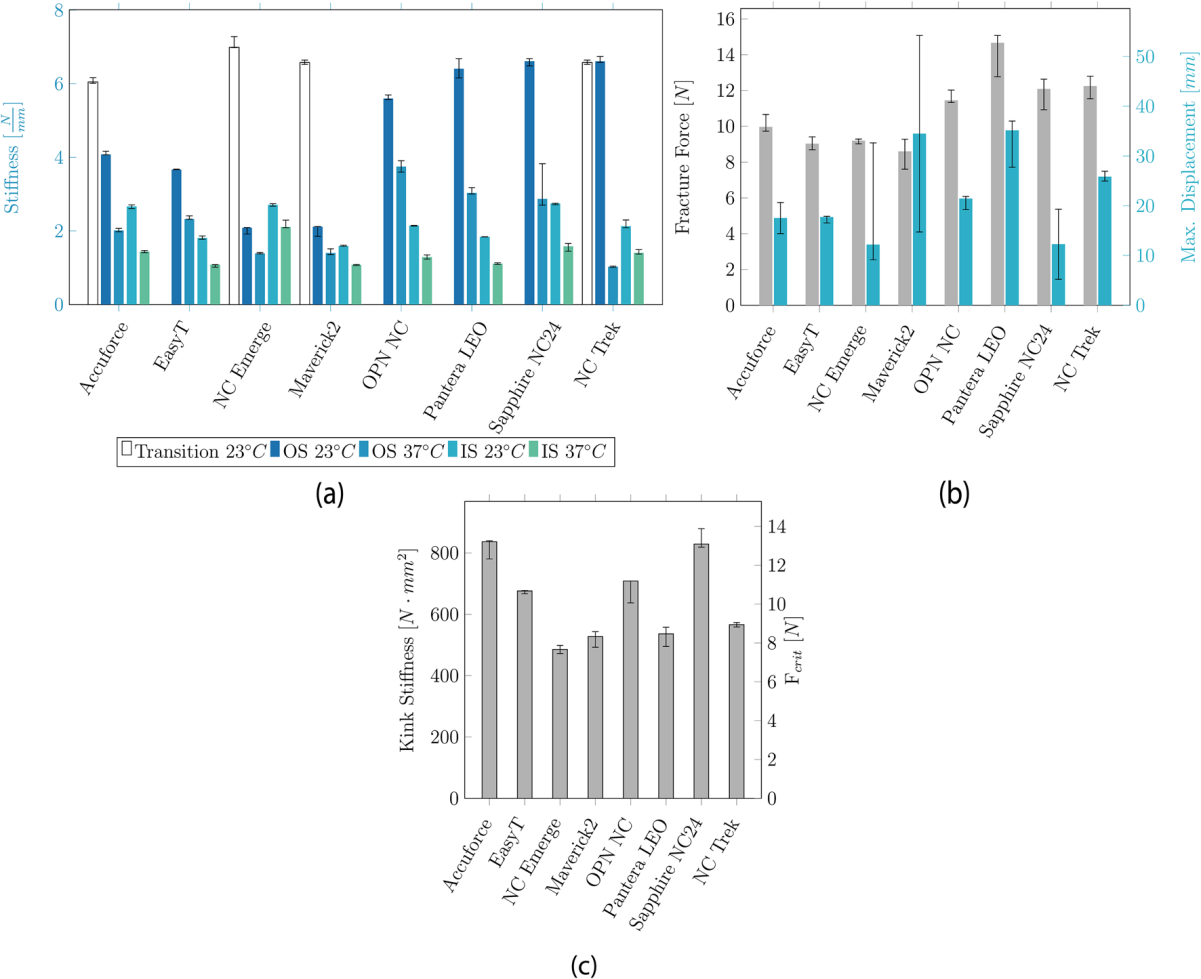
Results of the tensile and kink tests. **a** Median values and IQR of the tensile stiffness at 23°C and 37 °C. **b** Median values and IQR of the Fracture Force (gray) and Max. Displacement (blue) of the RX-Port measured at 37 °C. **c** Median values and IQR of the kink stiffness and the critical kink force $F_{crit}$ of the hypotube measured at 23 °C

The tensile stiffness obtained from the uniaxial tensile tests is shown in Fig. 3a. The transition part (from hypotube to RX-Port) is only shown for the Accuforce, NC Emerge, Maverick2, and the NC Trek. The Pantera LEO does not have this transition, and for the other catheters, the transition part showed the same behavior as the OS. An overview of the obtained yield points can be found in Appendix fig. 9

An increase in temperature causes the OS's stiffness and yield point to be reduced by 30–84% and 6–46 %, respectively. The IS’s stiffness and yield point are reduced by 22–45% and 15–32%, respectively.

Fig. 3b shows the tensile test result at 37 °C on the RX-Port. The RX-Port usually fractures at around 8.5–14.6 N and a displacement of 12–35 mm, approximately 34–100% of its start length. The force-displacement curves are depicted in Appendix figs. 10, 11, 12

Fig. 3c shows the kink stiffness and the critical kink force $F_{crit}$ of the hypotube. The kink stiffness and force range from 472–836 N·mm2 and 7.7–13.2 N, respectively.

### Bending Properties

**Fig. 4:**
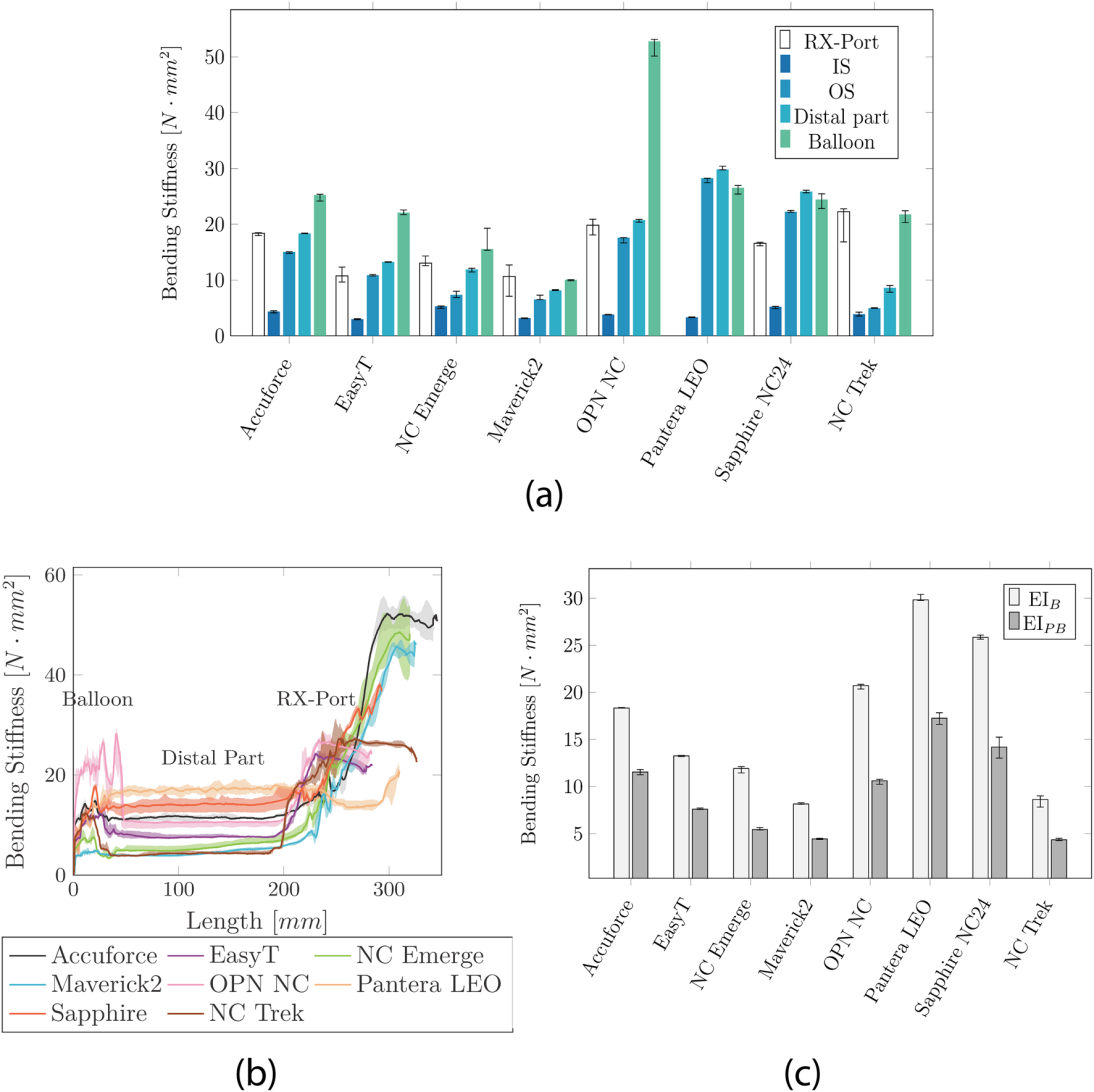
Median bending stiffness with IQR based on the 3-point bending test of each catheter. Measurement location: RX-Port, IS, OS, distal part, and the balloon. Measurement of the Bending Stiffness over the length of the distal catheter part. **a** Progression of the stiffness over the distal length. Lines show the median values, and shaded areas indicate the minimal and maximal measured values. **b** Results of the pulled bending tests show the bending stiffness of the balloon (approx. 0 – 40 mm), the bending stiffness of the distal part (approx. 40–200 mm), and the RX-Port (> 200 mm). Lines show the median values, and shaded areas indicate the minimal and maximal measured values. **c** Comparison of the bending stiffness of the distal part obtained from the 3-point bending and the pulled-bending test

Fig. 4 a shows the bending stiffness $EI_{B}$ measured on the RX-Port (10.7 – 22.2 N·mm^2^), the IS (3.1–5.3 N·mm^2^), the OS (4.9 – 28.2 N·mm^2^), the distal part (8.1–29.8 N·mm^2^), and the balloon (10.0–52.8 N·mm^2^). The distal part represents a measurement of the combined IS and OS. For each catheter, the IS overall shows the lowest bending stiffness. The bending stiffness of the OS is lowest for the NC Trek and the Maverick2 and highest for the Pantera LEO. The RX-Port of the Pantera LEO was not measured since it is located at the hypotube. The highest balloon stiffness was measured at the OPN NC. Since this test was performed on cut catheter parts, the bending stiffness of the tapered stiffening wire is not included.

The results of the pulled-3-point bending test are shown in Fig. 4 b. The balloon is measured from 0 to approximately 40 mm. The balloon is followed by a constant stiffness region, which indicates the distal part, including the IS and OS. The stiffness increases close to the RX-Port, indicating the stiffening wire.

The stiffness obtained from the pulled bending is compared to the 3-point bending test (cf. Fig. 4 c). The qualitative behavior is comparable. However, the stiffnesses obtained with the pulled-3-point bending test are lower.

### Push- and Trackability

A significant difference (*p* < 0.05) of track work measured in the LAD and the LCx was observed. Separate models to predict these values are needed. The subset regression for the pushability resulted in a significant model ($R_{adj}^{2} =$ 0.8056, *F* = 21.72, *p* value < 0.05) containing seven significant variables. All VIFs were below 10. The subset regression on the track work of the LAD resulted in a significant model ($R_{adj}^{2} =$ 0.9213, *F* = 52.19, *p* value < 0.05) with eight significant variables. All VIFs were below 10. However, from the investigation of the residuals, an extreme observation beyond Cook’s distance was found, necessitating a robust linear regression. A significant model ($R_{adj}^{2} =$ 0.8926, *F* = 49.48, *p* value < 0.05) was identified using subset regression on the track work of the LCx containing six significant variables. All the VIFs were below 10. However, an extreme observation beyond Cook’s distance was identified, necessitating a robust linear regression. The identified models are listed in Table 1.

**Table 1:** Multiple linear regression analysis of pushability and trackability

Variables	Pushability				Track work LAD robust linear regression			Track work LCX robust linear regression		
	Est.	Std. error	*t* value	*p* value	Est.	Std. Error	t value	Est.	Std. Error	*t* value
Intercept	− 59.776	14.26	− 4.192	2.51E−04	− 0.0274	0.0298	− 0.9199	− 0.406	0.0886	− 4.5819
CP					0.0918	0.0419	2.1905	0.5535	0.1072	5.1652
GCF	− 87.761	30.3	− 2.896	0.007						
GWF	39.641	16.331	2.427	0.022	− 0.1829	0.0763	− 2.3959	0.4576	0.2024	2.2612
BBS	− 0.746	0.092	− 8.088	8.32E−09	0.0038	0.0005	7.3067	0.0049	0.0015	3.1673
DBS					− 0.0039	0.0005	− 8.2225	− 0.0065	0.0011	− 5.9003
TS37	2.922	0.598	4.888	3.77E−05						
AF					0.039	0.0269	1.4481	0.2095	0.0789	2.6548
HD	228.07	37.437	6.092	1.43E−06						
Balloon elongation					− 0.0147	0.0074	− 1.9775			
TF					0.02	0.0058	3.4336			
Length GW	− 0.093	0.035	− 2.656	0.013						
Hypotube stiffness	− 0.036	0.009	− 4.233	2.24E−04						
Length stiffening wire					0.0002	0	6.3229	0.0003	0.0001	2.5465

CP: Crossing profile, GCF: Guide catheter friction, GF: Guidewire friction, BBS: Balloon bending stiffness, DBS: Distal bending stiffness, TS37: Stiffness of the distal shaft at 37 °C, AF: Artery friction, HD: Hypotube dimension, TF: Tip force

**Fig. 5:**
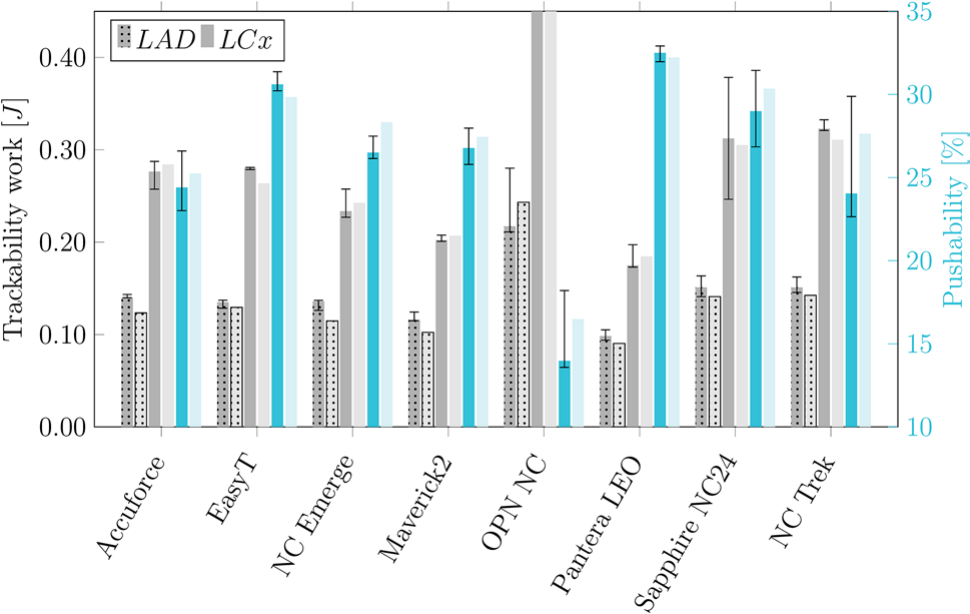
Measurement of the push- and trackability. Median values and IQR of the trackability work (gray) and pushability (blue). Light colored bars indicate the prediction from the multiple linear regression model

Fig. 5 shows the results of the push- and trackability tests and the prediction from the multiple linear regression model. The work required to insert the catheter into the LAD and LCx and to pull back the catheter is gray, and the pushability is blue. A higher pushability value means that more force is transmitted to the tip.

### Friction Characteristics

**Fig. 6:**
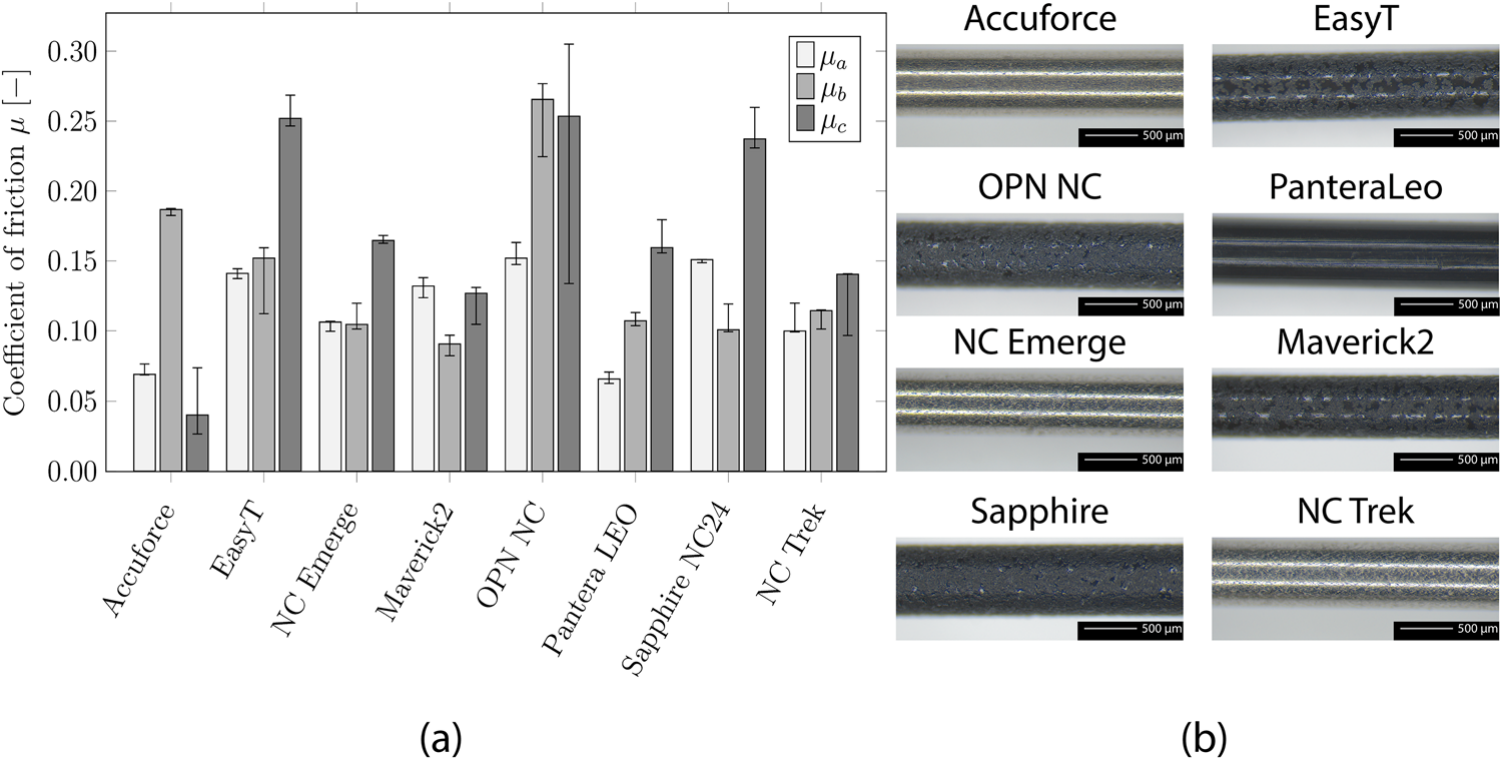
Frictional characteristics. **a** Median coefficients of friction with IQR measured at 37°C in water. $\mu_{a}$: between the hypotube and the GC, $\mu_{b}$: between the GW and the IS and $\mu_{c}$: between the OS and a porcine coronary artery. **b** Image of the surface of the hypotubes. (**b**) Microscopic images of the hypotube surface

The measured frictional coefficients are depicted in Fig. 6 a. The coefficient of friction $\mu_{a}$ and $\mu_{b}$ was found to be between 0.07–0.15 and 0.09–0.18, respectively. EasyT, OPN NC, and Sapphire NC24 showed the highest hypotube friction. The lowest hypotube friction was measured at the Accuforce, which has no visible coating on the hypotube, and the Pantera LEO, for which the coating is unknown.

The highest GW friction was found at the Accuforce and the EasyT. Compared to the other catheters, the friction variation is highest for EasyT. The lowest GW friction was found at the Maverick2. The other catheters showed similar values. The surfaces of the hypotube are shown in Fig. 6 b. It can be seen that very smooth surfaces like the Accuforce or Pantera LEO result in a low $\mu_{a}$. The surface of the EasyT, OPN NC, Maverick2, and Sapphire are comparable. This also becomes visible when comparing the $\mu_{a}$. The coefficient measured between th e OS and the porcine LAD $\mu_{c}$ was found to be between 0.04 (Accuforce) and 0.25 (EasyT and OPN NC).

### Torsional Stiffness

The highest torsional stiffness (0.87 mNmm/°) was measured for the OPN NC. In contrast, the Maverick2 (0.21 mNmm/°) and the NC Trek (0.25 mNmm/°) show the lowest torsional stiffness. A higher value means more torque can be transmitted from the hub to the tip, and therefore, the balloon is expected to do more rotations when rotating the hub. Furthermore, no significant difference between clockwise and counterclockwise rotation could be observed for the catheters, except for the OPN NC, where the counterclockwise rotation showed a slightly lower stiffness (clockwise: 0.87 mNmm/°)., counterclockwise: 0.82 mNmm/°). Detailed results are depicted in Appendix Fig. 17a ).

### Radio-opacity

It was already mentioned in ^1^ that the NC Trek uses tungsten-filled polymer markers. This results in a lower radio-opacity (8.6 mm Aluminum) than the other catheters that used the more common platinum-iridium markers. However, the radio-opacity of the Sapphire NC24 (9.3 mm Aluminum) is similar despite using the more common markers. The Pantera LEO showed the highest radio opacity (16.3 mm Aluminum). Detailed results are depicted in Appendix fig.17 ).

### Survey

#### Personal / General Questions

64% of the participants had more than ten years of experience, 9% had 5 – 10 years, and 27% had 2 – 5 years of experience as an interventional cardiologist.

A price of above 200$ was considered too expensive for 50% of the participants. 32% found a price between 100 and 200$ too expensive, and 9% a price ranging from 50 to 100$. Overall, the average price importance was 6.4 out of 10. Nevertheless, there exists a disparity in pricing between Switzerland and Germany. In Switzerland, a price exceeding $200 was deemed overly expensive, whereas in Germany, the range was between $100 and $200.

#### Usage

All catheters, except the OPN NC, are used for the treatment of lesions type A, B1, B2, and C equally (see Appendix fig. 18a). The OPN NC is mainly used for type C lesions and not used for type A and B1 lesions. The answer for type B2 lesions leaned towards neutrality. As an access site, no one is primarily using femoral access. Half of the cardiologists preferred radial access, whereas the other half used both accesses equally. The most frequently used GC size was 6 Fr, whereas some used 5 Fr for catheters like the Maverick2, Pantera LEO, and NC Trek. For the OPN NC, 7 Fr GC was also used.

In general, the catheters are used for primary dilatation of de-novo lesions (EasyT, Maverick2), Post-dilatation after stent implantation (all catheters except EasyT), In-stent re-stenosis PRE-dilation (Accuforce), In-stent re-stenosis POST-Dilatation (NC Emerge) or the treatment of chronic total occlusion (Sapphire NC24).

The most preferred catheter to perform the "Kissing-balloon technique" was the NC Trek. 4 out of 17 cardiologists would not use the OPN NC for the "Kissing-balloon technique". Most cardiologists would use the other catheters to perform the "Kissing-balloon technique".

Similarly, the participants agreed to use all catheters in tortuous vessels and to treat bifurcations, except the OPN NC (see Appendix fig. 18b).

#### Performance Characteristics:

The catheters are mainly used to treat moderate calcified lesions (see Appendix fig. 18c ). However, the OPN NC is used for moderate to severe cases and the Maverick2 for low to moderate cases.

The most reported complications with the catheter were the balloon deflation time and the balloon's burst (except EasyT and OPN NC). For the EasyT and the OPN NC, GW clamping after inflation was mentioned as a complication, and a torn shaft for the EasyT. No deflation, shaft burst, kink of the distal part, or hypotube kink was reported by any participant.

On the patient side, for nearly all catheters except the NC Trek, dissection was a recurring issue.

A PTCA balloon catheter's importance of torquability was rated 4 out of 10. However, this seems to depend on the interventional cardiologist's preferences since most answers were either a 1 out of 10 or a 7 – 10 out of 10.

The overall satisfaction of the Maverick2 is rated highest. In contrast, the overall satisfaction of the OPN NC was lowest. An overview of the performance satisfaction for each catheter is shown in Appendix fig.18d).

## Discussion

**Fig. 7:**
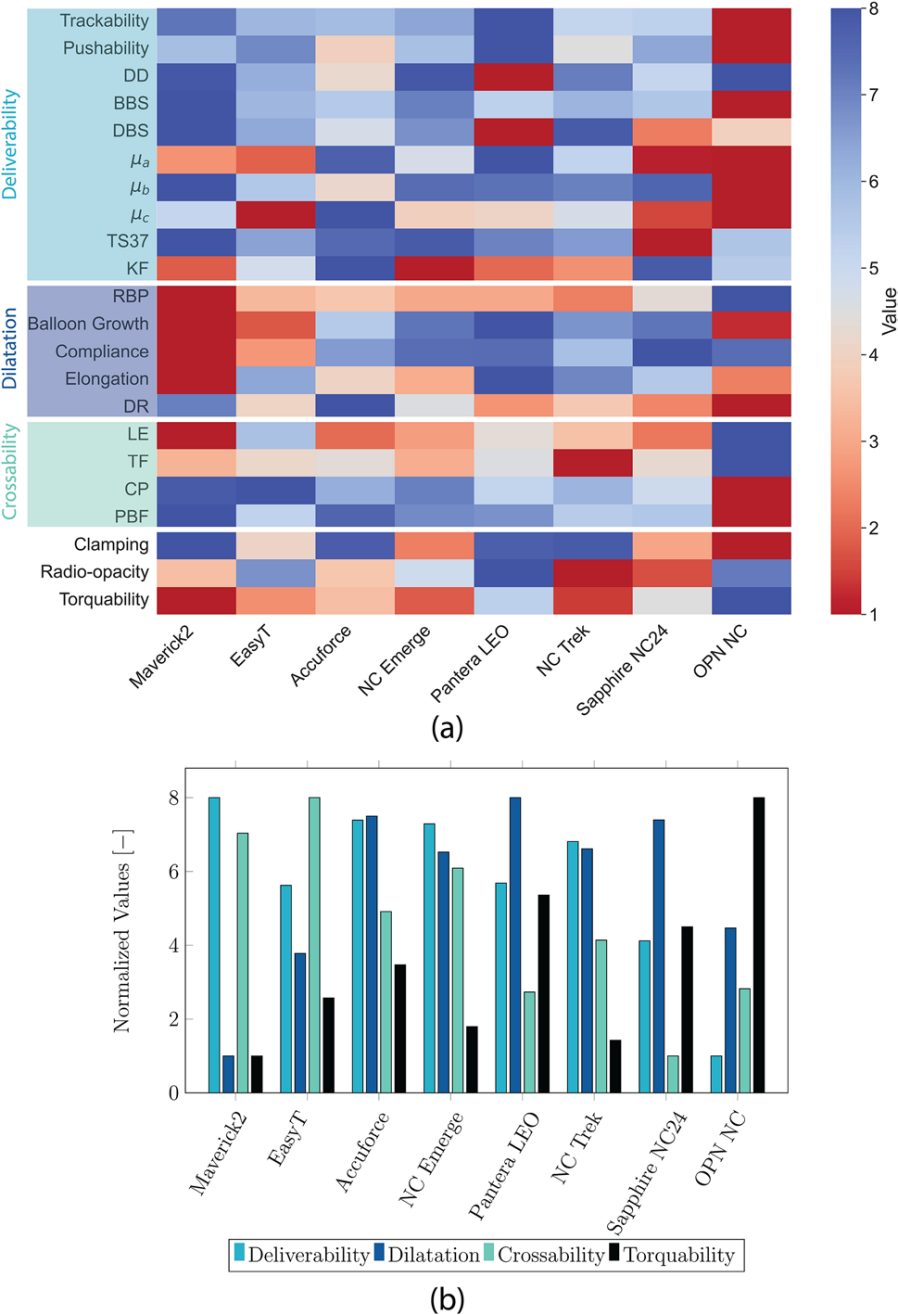
Overview of the tests performed on the catheters. The values represent normalized values, where the higher the value, the more beneficial it is. *DD* distal dimensions, *DBS* distal bending stiffness, $\mu_{a}$ guide catheter friction, $\mu_{b}$ guide wire friction,$\mu_{c} :$ artery friction, *TS* 37C tensile stiffness at 37° C, *KF* kink force, *BBS* balloon bending stiffness, *PBF* pull-back force, *CP* crossing profile, *TF* tip force, *LE* lesion entry, *DR* deflation rate, *RBP* rated burst pressure. (**a**) Heatmap comparing catheter performance: blue = highest, red = lowest. (**b**) Values of median deliverability, dilatation efficiency, and crossability

The data obtained during the analysis of the design of PTCA balloon catheters in our previous study ^1^ is included in the following for a better comparison between the catheters. The measured values are linear normalized and depicted in the heatmap in Fig. 7 a. The higher the value (blue), the better the performance; conversely, lower values (red) indicate poorer performance. For an overall estimation of the properties, the performance characteristics (deliverability, dilatation efficiency, crossability) are normalized (cf. Fig. 7 b).

Fig. 7 a shows that the Maverick2 offers the best deliverability and good crossability due to the soft balloon. However, the dilatation efficiency is the lowest compared to the other catheters. It has to be noted that the Maverick2 is an SC balloon catheter and is compared against NC balloons. Therefore, this result is to be expected. Furthermore, due to the relatively soft OS and IS, the torquability is reduced.

Whereas the Maverick2 is very flexible at the distal part, the distal stiffness of the EasyT is more comparable to the NC catheters. Even though the EasyT balloon offers a better dilatation efficiency than the Maverick2, it is still inferior to the other NC catheters. The measured crossability of the EasyT was the best.

The measured deliverability, dilatation efficiency, and torquability between the tested NC balloon catheters (NC Emerge, Accuforce, Pantera LEO, NC Trek, and Sapphire NC24) are comparable (cf. Fig. 7 a). On closer inspection, the deliverability is highest for the Accuforce and lowest for the Sapphire NC24, and the dilatation efficiency is highest for the PanteraLeo.

The best crossability was measured best for the NC Emerge and the lowest for the Sapphire NC24. The crossability of the Pantera LEO was reduced by the enlarged lesion entry profile, as shown in our previous research [27].

The OPN NC shows the lowest deliverability due to the very stiff balloon, the high coefficients of friction, and the low push-and-trackability. The dilatation efficiency was expected to be the best due to the unique twin-layered balloon [27]. Even though the RBP is high and the compliance low, the longitudinal elongation and overall percentage growth were high, resulting in less accurate balloon positioning and reduced dilatation efficiency. Enhanced tip properties contribute positively to crossability, promoting smoother navigation. Conversely, the large crossing profile negatively affects or even prevents the balloon from crossing. Even though the OPN NC's distal stiffness is lower than that of the Pantera LEO or Sapphire NC24 (cf. Fig. 4), the highest transmission between hub and balloon torque was measured. This could be because, in our setup, less torque was lost at the balloon. Although the OPN NC exhibits the highest torquability, it may be inferior to other catheters in terms of torque transmission within the coronary arteries. This limitation arises from factors such as the extensive profile, relatively stiff design, and high coefficient of friction, which can cause the balloon to become locked, impeding effective torque transmission. Therefore, it is assumed that torque is not solely based on the torsional properties of the device and may be governed by vessel tortuosity and lesion characteristics. Further research is necessary to understand this property fully.

**Fig. 8:**
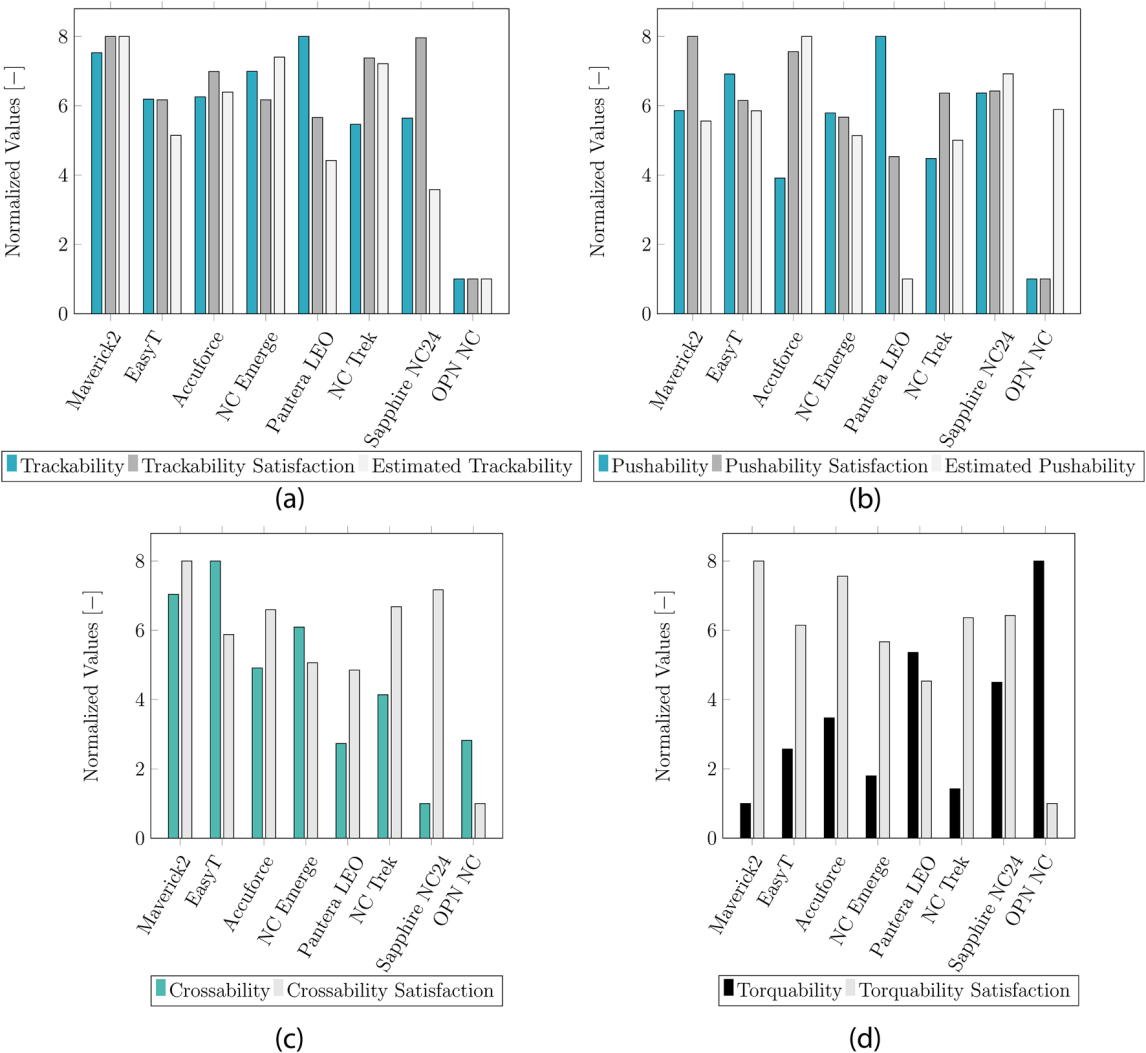
Overview of the performance characteristics of the individual catheters. **a** Normalization of the median trackability values in comparison to the results of the survey and an estimation of the trackability. **b** Normalization of the median pushability values in comparison to the results of the survey and an estimation of the pushability. **c** Values of the median crossability are based on a normalization of the surface area of the spider plot in comparison to the results of the survey. **d** Values of the median torquability in comparison to the results of the survey

The measured trackability, pushability, normalized crossability, and torqueability are compared with the survey findings (cf. Fig. 8).

Additionally, the trackability and pushability can be estimated based on the properties shown in Fig. 1 b) without performing simulated use-case tests. The trackability (cf. Fig. 8 a) was estimated based on the distal dimensions, the distal bending stiffness, and the frictional coefficients, and the pushability (cf. Fig. 8 b) was estimated based on the tensile stiffness, the kink force of the hypotube, the length of the IS, and the length of the tapered stiffening wire. Overall, the estimated trackability and pushability demonstrate closer alignment with the survey results than the outcomes derived from the simulated use-case tests. However, exceptions include the estimated trackability of the Sapphire NC24 and the estimated pushability of the OPN NC and the Pantera LEO.

The measured crossability of Sapphire NC24 shows the largest discrepancy from the survey result, where it was rated best among the NC balloons. Divergences from the survey data may be attributed to individual practitioner preferences. Cardiologists often favor a specific "work-horse" catheter, leveraging their extensive training to mitigate potential limitations. Tests on a stenotic model would help investigate crossability further.

Except for the Pantera LEO, the measured torquability exhibited a notable deviation from the survey results (cf. Fig. 8 d). This variance may stem from the fact that torquability is not a recognized feature of RX-PTCA balloon catheters among interventional cardiologists. Consequently, estimating torqueability poses a considerable challenge.

In general, the Maverick2 was rated highest in fulfilling clinical needs. In contrast to this, the OPN NC was generally rated the lowest in the survey for all properties (cf. Fig. 8 a – d). However, due to its unique features, it can treat lesions where other catheters cannot succeed. This shows that a comparison to the other PTCA balloon catheters lacks coherence since they are not used for this extent of calcification. A comparison to rotablation devices [31] would increase the significance regarding the dilatation efficiency.

In this investigation, all parameters were rated with the same importance. However, this might change depending on the interventional cardiologist, the patient, lesion characteristics, and the hospital. Therefore, an individual rating of the properties could help eliminate less important factors to bring the results of the measurements into a better context for the survey results. Furthermore, the number of participants in this survey is relatively low (*n* = 22), but it provides valuable insight.

Subsequently, the individual mechanical properties are discussed in more detail.

### Tensile and Kink Properties

During the tensile tests, no difference between the OS and the transition part was observed for the EasyT, OPN, Pantera LEO, and Sapphire NC24. Therefore, it is assumed that they use the same OS from the end of the hypotube until the balloon. Temperature elevation reduces the mechanical properties of all catheters, and this emphasizes the necessity of performing tests at body temperature.

The Sapphire NC24 showed a high scattering and brittle material behavior. A ductile behavior is desired to avoid fractures leading to stuck distal parts in the patient during an intervention. The same is true for the RX-Port. However, all catheters showed a ductile fracture behavior at the RX-Port with forces above 8 N.

For the hypotube, a high kink resistance is desired. A kinked hypotube can fracture, and thus, the balloon cannot be inflated or properly deflated anymore. Furthermore, the pull-back of the catheter becomes cumbersome. The main risk of kinking the hypotube is during insertion. However, a high kink stiffness indicates a high overall stiffness, which can negatively impact the trackability since the catheter cannot follow the curvature of the access route from the puncture site (radial or femoral) to the aortic root. High stiffness, in combination with high frictional forces, can cause a loss of push force.

### Bending Properties

The pulled-bending tests showed the stiffness progression over the catheter's distal part. However, it has to be noted that the measured value is always a mixture between three locations since the catheter bends at all three rolls. At the balloon segment, various peaks are visible, probably related to the increased stiffness of the radio-opaque markers.

Furthermore, different radio-opaque markers and crimping processes could be compared to find a suitable design. At the location of the distal part, the force remains constant. The constant force of the Sapphire NC24 indicates a constant cross-section of the stiffening wire. The transition between the balloon and the distal section of the Maverick is almost flat, indicating no stiffness increase. The distal section is followed by a progressive stiffness increase caused by the tapered stiffening wire, which is visible. No such increase is visible for the Pantera LEO due to the design. Close to the RX-Port of the Pantera LEO, the stiffness is reduced before it increases at the location of the skived hypotube. Again, specific peaks are visible at the location of the RX-Port. They can be caused due to the design and manufacturing of the RX-Port.

Comparing the stiffness values from the 3-point bending test to the pulled bending test shows a mismatch. This might be due to deviations between the theoretically derived and actual bending lines. However, the ratio varies between the catheters. Another influence can be the friction between the catheter and the setup.

The distal stiffness of the NC Trek is comparable to that of the Maverick2 and the NC Emerge. However, The Maverick2 has the lowest balloon bending stiffness and is rated with the best trackability. The balloon of the NC Emerge shows the second lowest balloon bending stiffness. However, the trackability was not rated very good. EasyT and NC Trek have comparable balloon stiffness. However, the distal part of the EasyT is stiffer.

### Frictional Characteristics

The obtained friction values cannot be compared to the data available in the literature since the frictional coefficient always depends on the environment. Comparing the obtained value of 0.012 from P. Wuensche et al. [29], a similar order of magnitude was found (0.09 – 0.019). The lower the friction, the smaller the losses over the length. This, in return, results in a better force transmission to the tip and an easier-to-advance catheter. The lowest coefficient of friction between the hypotube and the GC was found for Accuforce. It depends on the coating of the hypotube. The Maverick2 showed the lowest friction between the IS and the GW. The IS is usually manufactured from multi-layered tubing. The outer layer is selected for integrity and weldability to the OS and balloon. The inner layer is selected to reduce the friction between the GW. Based on the measurements between the porcine LAD and the OS, the Accuforce shows the lowest coefficient of friction. The friction coefficient between the LAD, the EasyT, and the OPN NC was the highest. It has to be noted that coronary artery disease does not appear in pigs, and therefore, this measurement is more comparable to healthy human coronary arteries than to diseased segments.

However, the catheter passes healthy coronary segments during the treatment; therefore, this value gives valuable insights into the performance of the catheters.

The current practice for testing the coating of the OS is to measure the frictional coefficient of the OS against a PTFE plate. However, using a different frictional partner to evaluate the coating might lead to an over/underestimation of the performance.

During these tests, similar values ranging from 0.04 to 0.25 were measured compared to the literature (0.046 [28] and 0.02 – 0.6 [30]). However, the actual OS of commercially available PTCA balloon catheters was tested against porcine coronary arteries for the first time.

### Push- and Trackability

The track test is a well-established test to measure the performance characteristics of balloon catheters. However, the proximally fixed GW can lead to deviations between the bench test and the intervention since the GW cannot be fixed inside the coronary arteries. Furthermore, the GC's support limits the force that can be applied to the catheter during an intervention. If the force applied to the catheter exceeds the support, the GC will be pushed out of the ostium. In the bench setup, the position of the GC is defined and cannot change during the test procedure. However, the setup can generate comparative measures between the catheters.

It was found that an increase in track work leads to a decrease in pushability. Since trackability is the inverse of track work, this relationship implies that a reduction in trackability results in a decrease in pushability. A less flexible catheter struggles to navigate through a tortuous vessel path. This increases the overall resistance from hub to tip, leading to greater energy losses and, ultimately, a decline in pushability.

According to the parameters from Table 1, an increase in the GCF, BBS, length of the GW lumen and kink stiffness of the hypotube results in a decrease in pushabililty. This is explained by an increase in friction between the GC and the PTCA balloon catheter, as well as an increase of the BBS or the kink stiffness, which results in a greater loss of transmitted forces when the PTCA balloon catheter has to navigate a tortuous path. Additionally, extending the GW lumen reduces the length of the metallic hypotube. Consequently, replacing a section of the hypotube, which efficiently transmits push forces due to its stiffness, with a less stiff segment results in a decrease in pushability. Additionally, enhancing the tensile stiffness of the distal section and increasing the dimensions of the hyptube improve its column strength, resulting in more effective force transmission. As mentioned earlier, Sirivella et al. [21] performed simulations on PTCA balloon catheters and found a strong correlation between the pushability and the HD.

As previously discussed, lower track work improves the trackability of a catheter. Therefore, a key design objective is to minimize track work. According to Table 1, increasing friction between the catheter and its surroundings leads to greater track work. This observation agrees with the simulations of Sirivella et al. [21], who also reported a strong influence of frictional properties on trackability. Similarly, increases in balloon size and stiffness negatively affect trackability. While an increase in HD results in better trackability for the LAD, the trackability in the LCx is negatively affected. The LAD is less tortuous than the LCx (refer to Fig. 6A). Consequently, the LCx demands more track work, necessitating greater catheter flexibility. This highlights the need for an optimal balance between flexibility and stiffness to meet the varying requirements of different catheter paths.

## Conclusion

Our study offers a detailed analysis of PTCA balloon catheters, assessing their performance across various critical parameters. The investigation showed that it is crucial to consider all mechanical and design properties during the catheter development. Trackability and pushability provide helpful insights into the performance of the PTCA balloon catheter. However, estimated values showed a better alignment with the survey results. The results further suggest that individual preferences of interventional cardiologists influence ratings.

While certain catheters demonstrate superior characteristics, individual strengths must be weighed against limitations. Even though our study provides valuable insights, further research incorporating realistic anatomy, including stenotic models, could deepen our understanding of catheter efficacy in clinical practice, aiding in more informed decision-making. Moreover, individualized property rating systems could offer tailored insights into catheter performance, ensuring relevance to clinical contexts.

## Figures and Tables

**Fig. 9:**
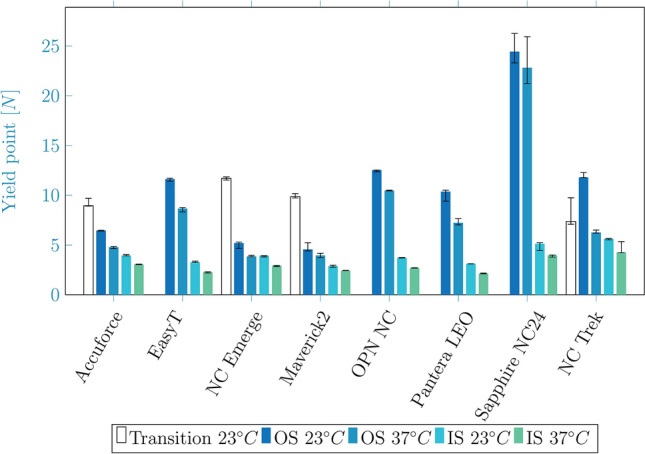
Median values of the yield point for the transition part, the OS, and the IS at 23 and 37 °C

**Fig. 10:**
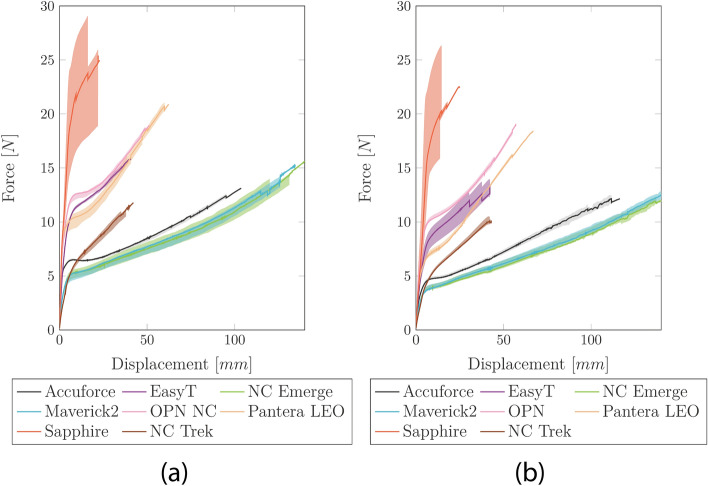
Median tensile force measured on the outer shaft: **a** at 23°C, **b** at 37 °C. The shaded areas indicate the scattering of the measurements (minimum and maximum values)

**Fig. 11:**
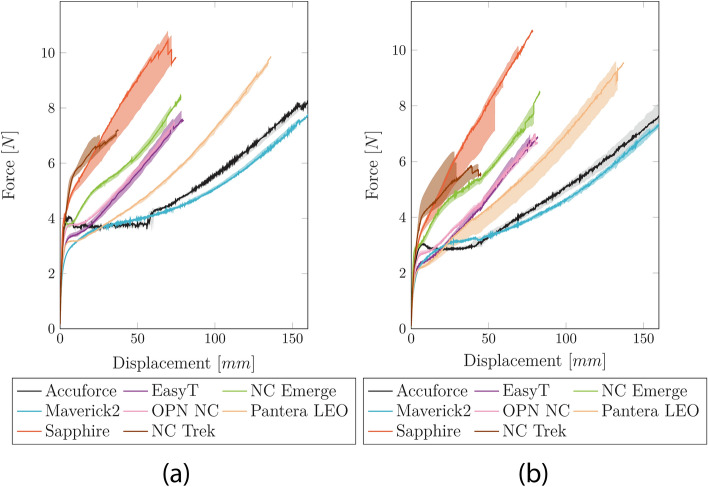
Median tensile force measured on the inner shaft: **a** at 23 °C, **b** at 37 °C. The shaded areas indicate the scattering of the measurements (minimum and maximum values)

**Fig. 12:**
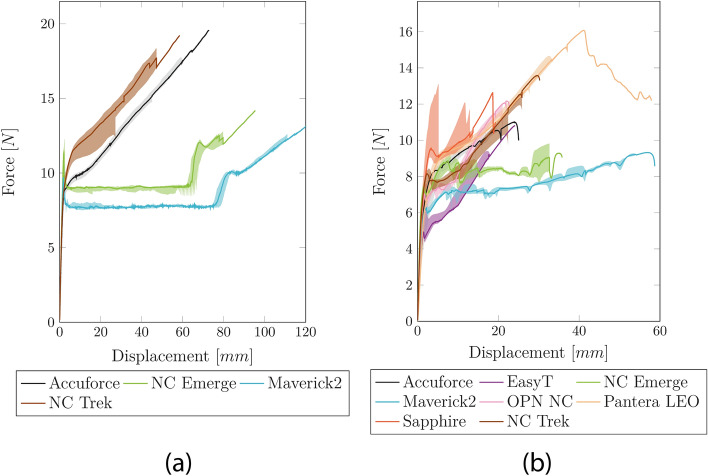
Median the tensile force measured: **a** at 23°C on the proximal outer shaft, **b** at 37°C on the RX-Port. The shaded areas indicate the scattering of the measurements (minimum and maximum values)

**Fig. 13:**
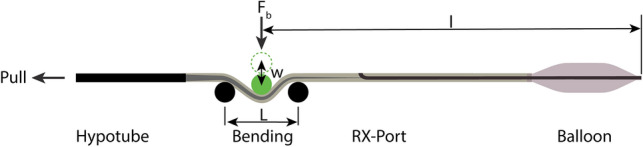
Schematic of the pulled-bending test to measure the bending stiffness along the distal part of a PTCA balloon catheter. Low friction pulley (green) and support (black) with a diameter of 4 mm and a distance of $L = 20{ }mm$

**Fig. 14:**
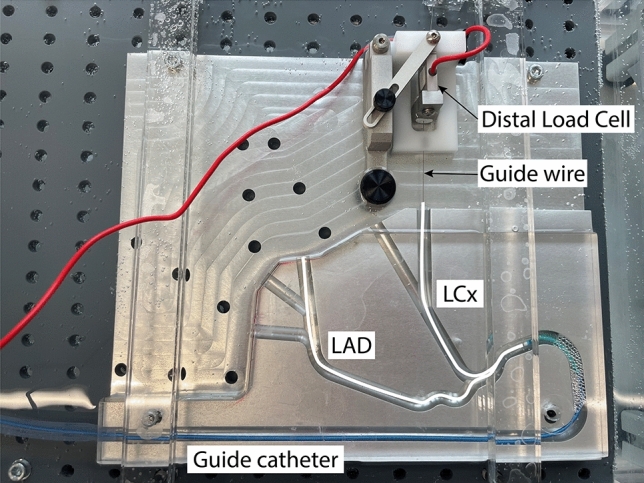
Anatomic track model (ASTM F2394) in a 37 °C water bath, including the LAD and the LCx coronary arteries, as well as the distal load cell used to evaluate the pushability. The proximal load cell is located on the left-hand side and is not shown in this picture

**Fig. 15:**
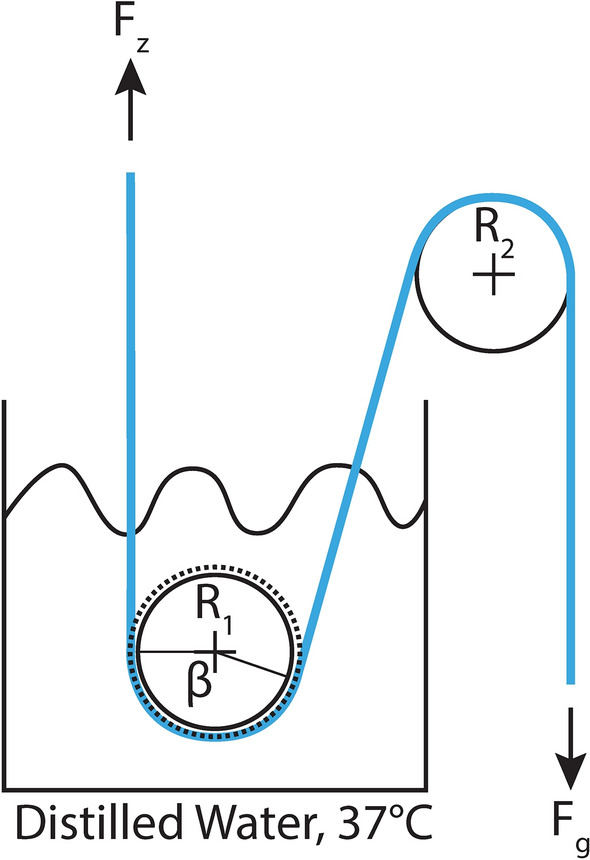
Schematic of the friction measurement in a bath with distilled water at a temperature of 37 °C. $F_{Z}$: tensile force, $F_{g}$: attached weight, $R_{1}$: lockable wheel with friction partner, $\beta$: contact angle, $R_{2}$: wheel with a diameter of 60 mm [29]

**Fig. 16:**
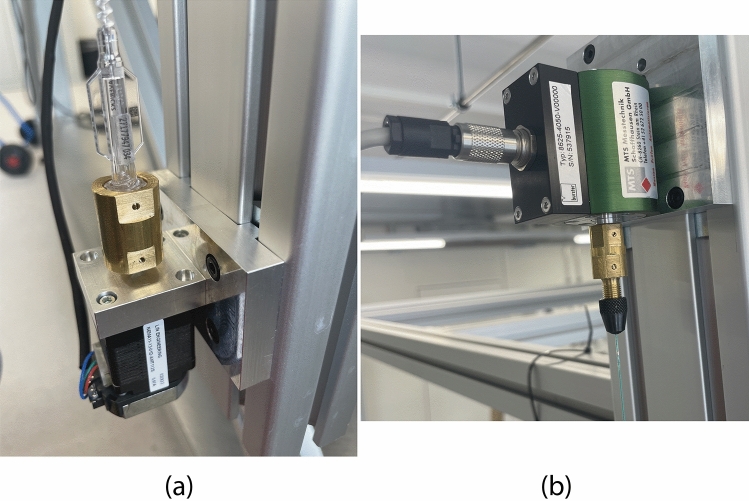
Setup for measuring the torsional stiffness of PTCA balloon catheters: **a** Stepper motor with the attached hub, **b** Torque sensor with the attached balloon

**Fig. 17:**
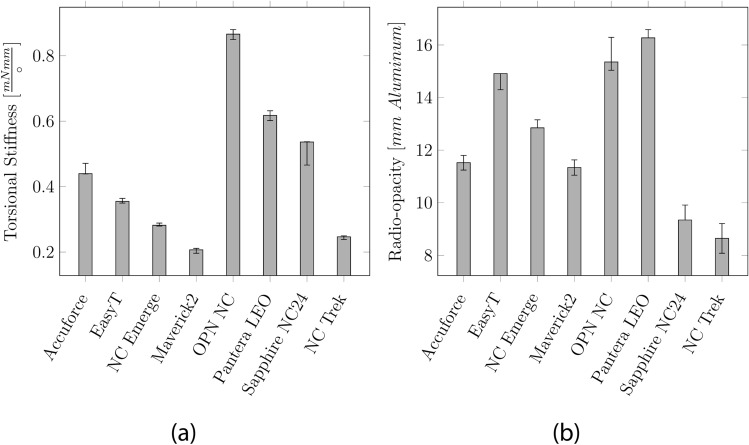
**a** Median values and IQR of the torsional stiffness. **b** Median values and IQR of the radio-opacity of the balloon markers

**Fig. 18:**
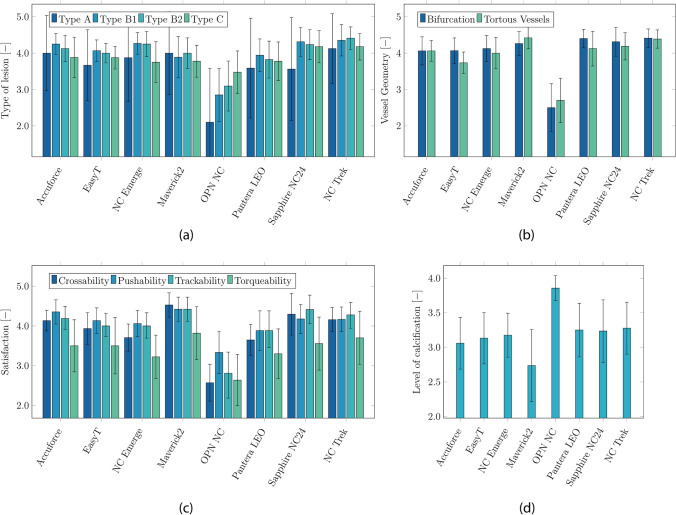
Average result and standard deviation of the questions asked during the survey. **a** Type of lesions, **b** Treated vessel geometry, **c** satisfaction regarding the performance characteristics: Crossability, pushability, trackability, and torqueability. 5: Very satisfying, 4: Somewhat satisfying, 3: Neither satisfying nor dissatisfying, 2: Somewhat dissatisfying, 1: Very dissatisfying, **d** Level of calcification

## Data Availability

The datasets used and/or analyzed during the current study are available from the corresponding author upon reasonable request.

## Appendix

### List of performed measurements and tested catheters.

See Table 2.

**Table 2:** Overview of the tests and used catheters.

Test procedure	Catheters 1–5	Catheters 6–10	Catheters 11−15
Measurement of the OD		x (no accessories)	
Measurement of the WT			x (no accessories)
Measurement of the ID			x (no accessories)
Fishmouthing test on the tip		x ( no accessories)	
Pushability	x (1 x GC, 8 x GW)		
Trackability	x (1 x GC, 8 x GW)		
Torquability	x (no accessories)		
Friction (GW)		x (1 x GC)	
Friction (GC)			x (1 x GW for two catheter types)
3-point-bending			x (no accessories)
Pulled bending			x (no accessories)
Tensile tests			x (no accessories)
Balloon elongation		x (1 x GC, 1 x GW)	
Pull-out forces		x (1 x GC, 1 x GW)	
Deflation-rate		x (1x GW)	
Radio-opacity			x (no accessories)

x indicates the use of 5 catheters, and brackets indicate used accessories. The measurements from the previous study are also included [27].

### Results of the tensile test

The following graph (Fig. 9) shows the measured yield points of the transition part between the hypotube and the distal part, the OS, and the IS measured at 23°C and at 37°C in a water bath.

This section shows the force-displacement curves of the tensile tests on catheter components like the OS (Fig. 9), IS (Fig. 10), and the transition part (Fig. 11a) measured at 23°C and in a water bath at 37°C. Furthermore, the tensile properties of the RX-Port measured in a water bath at 37°C are shown in Figs. 11b and 12.

### Setup for measuring the distal bending stiffness

The setup for measuring the distal bending stiffness over the whole length of the distal part is shown in Fig. 13.

The hypotube of the catheter is attached to a motorized linear stage (Igus® Inc., Cologne, Switzerland) with a travel distance of 400 mm. The catheter is placed on two supports (black) at a distance of $L = 20{ }mm$. A low friction pulley (green) is brought in contact with the catheter surface (zero position), followed by a displacement of $w = { }1.5{ }mm$. This travel distance was selected to avoid plastic deformation. After reaching the displacement, the linear stage pulls the catheter through the bending setup. During the pulling, the universal testing machine Shimadzu records the force $F_{b} \left( l \right)$ acting on the pulley over the distal length of the catheter $l$.

### Setup for measuring the push- and trackability

The GW is inserted into the anatomic track model (ASTM F2394) (cf. Fig. 14), which mimics the coronary arteries and is fixed at the proximal and distal ends to ensure its position. Furthermore, the GC is placed at the ostium of the coronary model.

### Setup for measuring the friction

A schematic of this setup to measure the friction on catheters is shown in Fig. 15.

For the measurement of the friction, a weight ($F_{g}$) is attached to a catheter or GW (blue). The catheter is guided over two wheels ($R_{1}$ and $R_{2}$). The frictional partner is attached to the lockable wheel $R_{1}$ which is immersed in a heated water bath at a temperature of 37 °C. The contact angle $\beta$ depends on the wheel diameter and the setup itself.

One test consists of two measurements: (1) assessment of the stiffness of the system with an unlocked $R_{1}$ ($F_{z1}$), (2) assessment of the frictional properties with a locked $R_{1}$ ($F_{z2}$).

### Setup for measuring the torsional stiffness

Motion control of the stepper motor (cf. Fig. 16 a) is implemented by the ROB-12859 motor driver (SparkFun Electronics, Niwot, Colorado, USA), set to 16 micro-steps, and the HMF2550 Arbitrary Function Generator (Rohde & Schwarz USA, Columbia, Maryland, USA). The function generator produces a square-wave signal (0-5 V) for the motor driver that specifies the stepwise movement of the stepper motor. Manipulating the frequency and number of steps allows precise motor speed and rotation angle control. Without an encoder, the stepper motor executes 200 full steps per revolution, which are further subdivided into 16 micro-steps each. A micro-step may be lost in a worst-case scenario, causing the motor to transition abruptly to a full step. Consequently, in the worst case, this results in a rotation of 360/200 = 1.8°. However, this error is less likely due to the low torque involved. The high-precision torque sensor 8625 4050 (cf. Fig. 16 b) has an integrated amplifier for an output range of + − 10V

### Results of the friction, torque and radio-opacity

See Fig. 17.

### Overview of the survey questions and results.

See Table 3.

**Table 3:** Overview of the questions and the respective question types asked in the survey

Question	Type
Current position	Text-based, optional
Hospital	Text-based, optional
Years of experience	Multiple choice
Country	Text-based, optional
Would you treat a type A lesion with this PTCA balloon catheter?	Likert scale, strongly agree to strongly disagree.
Would you treat a type B1 lesion with this PTCA balloon catheter?	Likert scale, strongly agree to strongly disagree.
Would you treat a type B2 lesion with this PTCA balloon catheter?	Likert scale, strongly agree to strongly disagree.
Would you treat a type C lesion with this PTCA balloon catheter?	Likert scale, strongly agree to strongly disagree.
Type of lesion treated	Multiple choice
Which access do you prefer with this PTCA balloon catheter?	Multiple choice
Level of calcification until which you would use the PTCA balloon catheter?	Multiple choice, no calcification to severe calcification
Would you use the PTCA balloon catheter to treat bifurcations?	Likert scale, strongly agree to strongly disagree.
Would you use the PTCA balloon catheter to treat lesions in tortuous vessels?	Likert scale, strongly agree to strongly disagree.
Would you perform the "Kissing technique" with the PTCA balloon catheter?	Likert scale, strongly agree to strongly disagree.
Which Guidecatheter do you prefer for the PTCA balloon catheter?	Multiple choice, GC sizes (5Fr – 8 Fr)
How well is the crossability of the PTCA balloon catheter?	Likert scale, very satisfying to very dissatisfying
How well is the trackability of the PTCA balloon catheter?	Likert scale, very satisfying to very dissatisfying
How well is the pushability of the PTCA balloon catheter?	Likert scale, very satisfying to very dissatisfying
How well is the torqueability of the PTCA balloon catheter?	Likert scale, very satisfying to very dissatisfying
Are there some recurring complications with the PTCA balloon catheter? (Patient)	Multiple choice
Are there some recurring complications with the PTCA balloon catheter? (Catheter)	Multiple choice
How important would you rate the torquability of a PTCA balloon catheter?	Scale from 1 to 10 (10 is very important)
How important would you rate the price of a PTCA balloon catheter?	Scale from 1 to 10 (10 is very important)
Above what price do you consider a PTCA balloon catheter too expensive?	Multiple choice, optional
Is there a point (or points) where you still see potential improvements in PTCA balloon catheters or the procedure? (Optional)	Text-based

The results of the survey questions are shown in Fig. 18.

## Declarations

### Citation Diversity Statement

Recent work in several fields of science has identified a bias in citation practices such that papers from women and other minority scholars are undercited relative to the number of papers in the field [32–35]. We recognize this bias and have worked diligently to ensure that we are referencing appropriate papers with fair gender and racial author inclusion.
